# Supplementary material for: Metagenome-Wide Analysis of Rural and Urban Surface Waters and Sediments in Bangladesh Identifies Human Waste as a Driver of Antibiotic Resistance
Source: mSystems. 2021 Jul 13;6(4):e00137-21. doi: 10.1128/mSystems.00137-21 (PMC8407206; doi:10.1128/mSystems.00137-21)
Supplement: TABLE S1 [file msystems.00137-21-st001.pdf]

Sample[a]	pH	Dissolved Oxygen (%)	T (°C)	Surface water type	Fish[b]	Antibiotics used[c]	Date collected
**WAM1**	7.8	76.8	26.4	Commercial aquaculture	Shing (fingerlings)	C	08/05/2018
**WAM2**	7.7	89.7	27.9	Commercial aquaculture	Shing	E, S, T	08/05/2018
**WAM3**	7.6	73.1	27.8	Commercial aquaculture	Koi	C, O	08/05/2018
**WAM4**	7.6	37.9	27.1	Commercial aquaculture	Pabda	C, E, S, T	08/05/2018
**WAM5**	7.3	73.8	27.3	Commercial aquaculture	Pangas	C, S	08/05/2018
**WAM6**	7.1	59.9	28.0	Commercial aquaculture	Pangas, Shing	C, E, S, T	08/05/2018
**WCM1**	7.7	70.6	26.5	Household pond	NA	NA	08/05/2018
**WCM2**	8	97.1	26.8	Household pond	NA	NA	08/05/2018
**WCM3**	9.2	126.8	27.0	Household pond	NA	NA	08/05/2018
**WAS1**	7.5	68.3	32.1	Commercial aquaculture	Rui, Catla, Mrigal	E	11/06/2018
**WAS2**	8.4	112	32.1	Commercial aquaculture	Rui, Catla, Mrigal	C, E, S	11/06/2018
**WAS3**	8.3	102.6	34.2	Commercial aquaculture	Rui, Catla, Mrigal	C	11/06/2018
**WAS4**	8.9	135.2	35.9	Commercial aquaculture	Rui, Catla, Mrigal	E, S	11/06/2018
**WAS5**	7.8	66.8	35.7	Commercial aquaculture	Rui, Catla, Mrigal	C, S, T	11/06/2018
**WCS1**	8.1	85.2	31.6	Commercial aquaculture (no antibiotic use)	Rui, Catla, Mrigal (fingerlings)	NA	11/06/2018
**WCS2**	7.4	44	31.3	Commercial aquaculture (no antibiotic use)	NA	NA	11/06/2018
**WCS3**	7.5	69.3	34.1	Commercial aquaculture (no antibiotic use)	NA	NA	11/06/2018
**WD1**	7.1	6	26.8	Urban lake	NA	NA	11/05/2018
**WD2**	7.4	15.4	28.0	Urban lake	NA	NA	11/05/2018
**WD3**	7.6	67.7	32.5	Urban pond	NA	NA	11/05/2018
**WD4**	7.4	78.4	32	Urban lake	NA	NA	11/05/2018
**WD5**	7.8	76	30.5	Urban pond	NA	NA	11/05/2018
**WD6**	7.1	12.3	28.2	Urban river	NA	NA	18/05/2018
**WD7**	7.1	9.5	28.3	Urban river	NA	NA	19/05/2018

[a] Surface water samples are coded as follows. W identifies them as water samples, the second letter identifies whether the samples were collected in rural settings (A: aquaculture, C: control, non-aquaculture) or urban sites (D: Dhaka). In the samples collected at rural sites, the region is indicated with the final letter (M: Mymensingh, S: Shariatpur).

[b] Binomial names: Shing: *Heteropneustes fossilis*, Koi: *Anabas cobojius,* Pabda: *Callichrus pabda*, Pangas: *Pangasius pangasius*, Rui*: Labeo rohita*, Catla: *Catla catla*, Mrigal: *Cirrhinus cirrhosis*; NA: not applicable

[c] C: ciprofloxacin, E: erythromycin, S: sulfadiazine, T: trimethoprim, O: oxytetracycline; NA: not applicable.
