# Supplementary figures and images for: Metagenome-Wide Analysis of Rural and Urban Surface Waters and Sediments in Bangladesh Identifies Human Waste as a Driver of Antibiotic Resistance
Source: mSystems. 2021 Jul 13;6(4):e00137-21. doi: 10.1128/mSystems.00137-21 (PMC8407206; doi:10.1128/mSystems.00137-21)

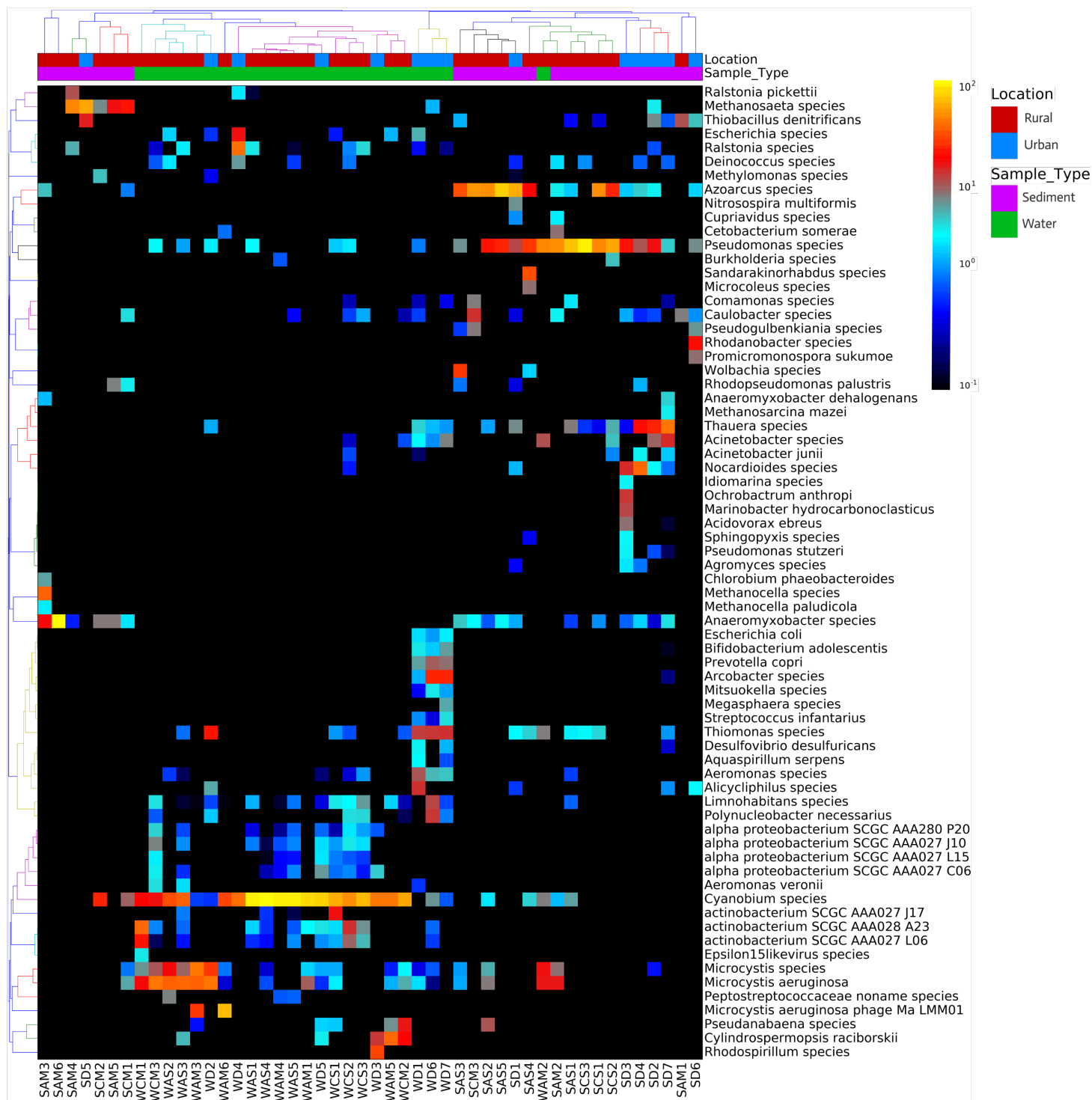

Supplement: FIG S1 [file msystems.00137-21-sf001.pdf]

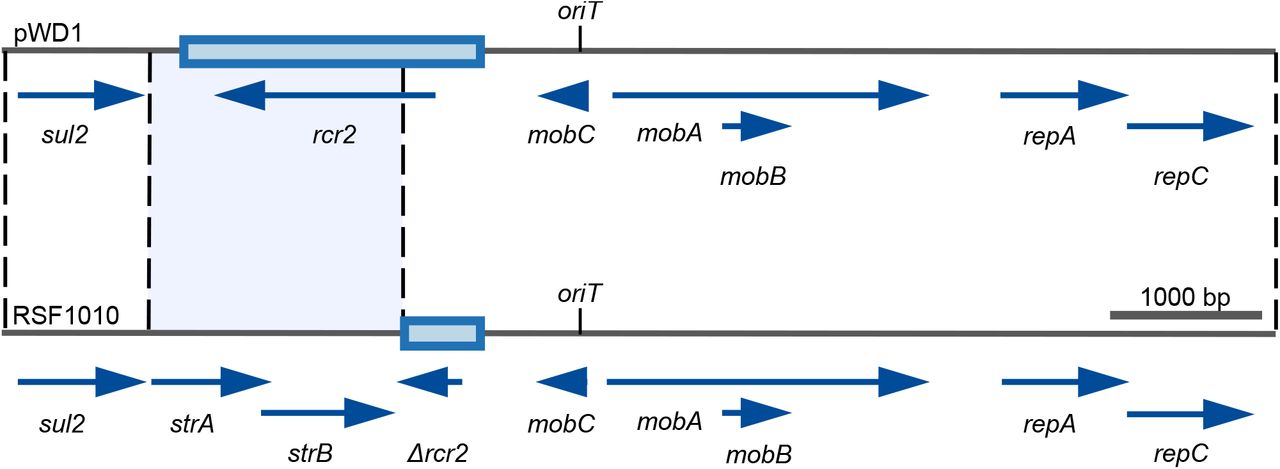

Supplement: FIG S2 [file msystems.00137-21-sf002.jpg]
