# Supplementary material for: Metagenome-Wide Analysis of Rural and Urban Surface Waters and Sediments in Bangladesh Identifies Human Waste as a Driver of Antibiotic Resistance
Source: mSystems. 2021 Jul 13;6(4):e00137-21. doi: 10.1128/mSystems.00137-21 (PMC8407206; doi:10.1128/mSystems.00137-21)
Supplement: TABLE S2 [file msystems.00137-21-st002.pdf]

Sample	Contig ID[a]	Rep type	Length (bp)	Coverage (%)[b]	Identity (%)[b]
**WCM1**	k141_156157	Col156	5212	100	94.81
	k141_206349*	Col(BS512)	2254	100	100
	k141_35625	Col8282	3712	100	80.88
**WD1**	k141_304072*	IncQ1	8676	100	100
	k141_320207	IncQ	1420	51.33	88.31
	k141_593572	repUS43	1262	50.41	96.05
	k141_711213	IncQ1	24244	78.39	77.48
	k141_728573	Col(pWES)	1941	93.26	80.95
**WD2**	k141_452869	IncQ	2296	78.39	77.48
**WD7**	k141_315908	IncP6	1166	100	99.63
	k141_77466	IncQ	4072	78.39	77.48

[a] Plasmid contig which could be circularised.

[b] Coverage and identity are of the closest *rep* gene in the PlasmidFinder database.
