# Supplementary material for: Metagenome-Wide Analysis of Rural and Urban Surface Waters and Sediments in Bangladesh Identifies Human Waste as a Driver of Antibiotic Resistance
Source: mSystems. 2021 Jul 13;6(4):e00137-21. doi: 10.1128/mSystems.00137-21 (PMC8407206; doi:10.1128/mSystems.00137-21)
Supplement: TABLE S3 [file msystems.00137-21-st003.pdf]

Sample	Contig	Closest BLAST match in Genbank	Bacterial host of closest match	Identity (%)	Coverage (%)
**WCM3**	k141_292096	pPm14C18	*Proteus mirabilis*	99.88	100.00
**SD1**	k141_106889	pSCU-397-2	*Escherichia coli*	99.11	99.00
	k141_227167	pAN70-1	*Alcaligenes faecalis*	99.96	100.00
	k141_704017*	pNFYY023-1	*Comamonas testosteroni*	99.94	91.00
	k141_836485	pG5A4Y217	*Escherichia coli*	99.34	58.00
	k141_99417	pKP14812-MCR1	*Klebsiella pneumoniae*	97.57	40.00
**WD1**	k141_128693	p33	*Escherichia coli*	100.00	76.00
	k141_134061	Unnamed plasmid	*Butyricimonas faecalis*	89.14	28.00
	k141_139895	pCP8-3-IncFIB	*Escherichia coli*	95.43	99.00
	k141_160447*	pCAV1335-92	*Klebsiella oxytoca*	98.76	100.00
	k141_205613	p1	*Klebsiella pneumoniae*	99.76	90.00
	k141_256831*	pGENC284	*Enterobacter hormaechei* subsp. *Xiangfangensis*	100.00	100.00
	k141_304072	pSL7202-3	*Salmonella enterica* subsp. *enterica* serovar *Typhimurium*	99.98	81.00
	k141_344452	pRWC72a	Uncultured bacterium	98.60	100.00
	k141_377411*	pUCLAOXA232-5	*Klebsiella pneumoniae*	100.00	41.00
	k141_391604	pTZC1	*Cutibacterium acnes*	98.57	91.00
	k141_44508	pJF-786	*Enterobacter cloacae*	99.83	86.00
	k141_467424*	pYH12207-3	*Acinetobacter piscicola*	100.00	92.00
	k141_510896	pG5A4Y217	*Escherichia coli*	99.46	51.00
	k141_604491	pSTN0717-64-1	*Enterobacter hormaechei*	99.75	100.00
	k141_719363	pSAN1-06-0624	*Salmonella enterica* subsp. *enterica* serovar *Anatum*	100.00	100.00
	k141_719904	pAeme6	*Aeromonas media*	99.77	100.00
	k141_804869	pMRGN207	*Escherichia coli*	99.59	70.00
	k141_881861	pBS228	*Pseudomonas aeruginosa*	99.94	92.00
	k141_903595	pWCX23_1	*Aeromonas hydrophila*	99.87	100.00

	k141_91069	pPN3F2_1	*Shewanella aestuarii*	99.23	68.00
**SD2**	k141_324783	pEC422_1	*Escherichia coli*	99.91	81.00
	k141_325504	Unnamed plasmid	*Klebsiella michiganensis*	100.00	33.00
	k141_343850	RW109	*Pseudomonas aeruginosa*	99.92	58.00
	k141_461478*	pAN70-1	*Alcaligenes faecalis*	100.00	100.00
	k141_701410	pGENC284	*Enterobacter hormaechei* subsp. *Xiangfangensis*	100.00	100.00
	k141_743709	pN1566_2	*Salmonella enterica* subsp. *enterica* serovar *Schwarzengrund*	99.77	100.00
**WD2**	k141_109523	pVB82_1	*Acinetobacter baumannii*	99.94	92.00
	k141_113493*	pYH12207-3	*Acinetobacter piscicola*	99.93	78.00
	k141_500987	p24358-1	*Salmonella enterica* subsp. *enterica* serovar *Bredeney*	99.86	100.00
	k141_723703*	pNFYY023-1	*Comamonas testosteroni*	100.00	99.00
	k141_740211	pVCGX2	*Vibrio campbellii*	99.94	100.00
	k141_740911	pMH17-012N_3	*Citrobacter freundii*	99.19	95.00
	k141_76560	p3	Novosphingobium sp. *ES2-1*	99.93	72.00
	k141_777886	p1681-tetX	*Empedobacter falsenii*	98.40	98.00
**SD3**	k141_1668271	pOXA58_010030	*Acinetobacter defluvii*	99.82	100.00
	k141_625842	pCF39S	*Pseudomonas aeruginosa*	99.94	99.00
**SD4**	k141_546527	pEI-2234-3	*Edwardsiella ictaluri*	61.00	100.00
**SD5**	k141_806265	pG5A4Y217	*Escherichia coli*	99.78	74.00
**WD5**	k141_583117	pGENC284	*Enterobacter hormaechei* subsp. *Xiangfangensis*	99.81	59.00
**SD6**	k141_104572	pAH01-4	*Escherichia coli*	99.74	100.00
**WD6**	k141_124684	p116753-FIIK	*Klebsiella pneumoniae*	100.00	82.00
	k141_158642*	pPm14C18	*Proteus mirabilis*	99.90	51.00
	k141_173719	pWP7-S18-ESBL-04	*Klebsiella* sp. *WP7-S18-ESBL-04*	99.97	72.00
	k141_243506*	pYPR31	*Providencia rettgeri*	100.00	100.00
**SD7**	k141_100623	p63039	*Myroides odoratimimus*	99.89	100.00
	k141_204766	pKP20194a-p3	*Klebsiella pneumoniae*	100.00	100.00

	k141_225349	p1	*Neisseria gonorrhoeae*	98.74	100.00
	k141_239404	p24358-1	*Salmonella enterica* subsp. *enterica* serovar *Bredeney*	99.97	99.00
	k141_24536	pA2293-Ct2	*Klebsiella pneumoniae*	99.64	100.00
	k141_270502	pNA6	Uncultured bacterium	99.93	30.00
	k141_333949	pLraf_19_5_1	*Lactococcus raffinolactis*	94.90	37.00
	k141_383255	pHNCF11W-130kb	*Escherichia fergusonii*	100.00	100.00
	k141_393768	pVCGX2	*Vibrio campbellii*	99.83	100.00
	k141_451891	pRErm46	*Rhodococcus hoagii*	99.00	89.00
	k141_464290	pC16KP0065-1	*Klebsiella pneumoniae*	100.00	39.00
	k141_479837	pEI-2234-3	*Edwardsiella ictaluri*	99.90	100.00
	k141_556637	pRGRH0399	Uncultured bacterium	94.84	80.00
	k141_557329	pHDC14-2.133K	*Enterococcus hirae*	98.45	88.00
	k141_569663*	pNFYY023-1	*Comamonas testosteroni*	99.98	80.00
	k141_574437	Plasmid 2	*Salmonella enterica* subsp. *enterica* serovar *Typhi*	99.44	96.00
	k141_94024*	pAb-C63_1	*Acinetobacter baumannii*	99.95	79.00
**WD7**	k141_113036	pEI-2234-3	*Edwardsiella ictaluri*	100.00	100.00
	k141_119966	pHNCF11W-130kb	*Escherichia fergusonii*	100.00	100.00
	k141_139664	pMS2H5VEB-1	*Klebsiella pneumoniae*	99.88	95.00
	k141_16407	pBS228	*Pseudomonas aeruginosa*	99.79	71.00
	k141_174331	pRSB222	Uncultured bacterium	97.35	83.00
	k141_262055	pG5A4Y217	*Escherichia coli*	90.98	85.00
	k141_323094	pN1566_2	*Salmonella enterica* subsp. *enterica* serovar *Schwarzengrund*	100.00	100.00
	k141_337873	pMH17-012N_3	*CItrobacter freundii*	98.83	92.00
	k141_362572	pALTS33	Uncultured bacterium	93.81	54.00
	k141_38198	pTet	*Campylobacter jejuni* subsp. *jejuni*	99.89	100.00
	k141_389765	pCAP01	*Capnocytophaga ochracea*	89.11	35.00
	k141_390832	pMR0211	*Providencia stuartii*	99.96	95.00
	k141_47250	pE211-2	*Enterococcus faecalis*	99.41	100.00

	k141_51571*	p3iANG	*Vibrio cholerae*	97.91	85.00
	k141_5646*	pYH12207-3	*Acinetobacter piscicola*	100.00	96.00
	k141_7584	p63039	*Myroides odoratimimus*	99.89	26.00
	k141_93260*	pSY153-MDR	*Pseudomonas putida*	99.97	96.00
	k141_98708	p345-185	*Vibrio harveyi*	94.32	77.00
**SAS1**	k141_249579	p3	*Novosphingobium* sp. *ES2-1*	99.92	85.00
**WAS1**	k141_49220	p4130-KPC	*Pseudomonas aeruginosa*	99.93	100.00
**SAS2**	k141_781158	pEI-2234-3	*Edwardsiella ictaluri*	100.00	100.00
**WAS3**	k141_1155247	p3	*Novosphingobium* sp. *ES2-1*	90.90	92.00
**WAM1**	k141_388101	pP72_e	*Phaeobacter inhibens*	98.95	99.00
	k141_448754	pEI-2234-3	*Edwardsiella ictaluri*	99.65	100.00
**WAM3**	k141_457349	pCF39S	*Pseudomonas aeruginosa*	100.00	88.00
**WAM6**	k141_67005	pHH2-227	Uncultured bacterium	99.94	99.00
